# Increased anti-nucleocapsid secretory IgA and consumption of complement component 3 in post-COVID syndrome patients

**DOI:** 10.3389/fimmu.2026.1822171

**Published:** 2026-05-14

**Authors:** Zhiwen Hai, Weihua Yang, Azam Ghazi, Amalia Buitrago, Patricia Marín-García, Isabel G Azcárate, Alba González-Escalada, Nineth Rossi, Javier Benítez-Cruz, Iván Estévez-Benito, Agustín Tortajada, José R. Regueiro, José M. Bautista, Narcisa Martinez-Quiles

**Affiliations:** 1 Department of Immunology, Ophthalmology and ENT, Complutense University School of Medicine, Universidad Complutense de Madrid, Avda de la Complutense, Madrid, Spain; 2 Department of Biochemistry and Molecular Biology & Area of Infectious Diseases and AIDS, Research Institute Hospital 12 de Octubre (imas12), Madrid, Spain; 3 Universidad Complutense de Madrid, Ciudad Universitaria, Madrid, Spain; 4 Immunology section, Fac. de CC. de la Salud, Departamento de Especialidades Médicas y Salud Pública, Universidad Rey Juan Carlos (URJC), Alcorcón, Spain; 5 Microbiology section, Fac. de CC. de la Salud, Departamento de Especialidades Médicas y Salud Pública, Universidad Rey Juan Carlos (URJC), Alcorcón, Spain; 6 Research Institute Hospital 12 de Octubre (imas12), Madrid, Spain

**Keywords:** anti-Nucleocapsid salivary IgA, C3 consumption, candidate biomarkers, circulating immune complexes, complement system, COVID-19 vaccines and reinfections, long covid, post-COVID syndrome

## Abstract

**Introduction:**

Post-COVID syndrome represents a major global health challenge which is characterized by immune dysregulation, although many aspects of the immune response remain incompletely understood, particularly the antibody response and the role of the complement system. We previously studied a post-COVID syndrome cohort in comparison to a COVID-recovered control cohort from Comunidad de Madrid (Spain) and found that post-COVID syndrome patients exhibited readily detectable serum anti-Nucleocapsid antibodies while showing deficient antibody production against the full-length Spike, despite maintaining a well-preserved anti-receptor binding domain (RBD) response.

**Methods:**

In the present study, we quantified anti-Nucleocapsid secretory immunoglobulin A (sIgA) in saliva and analyzed selected key components of the complement system, including C3, C4, factor B (FB), factor H (FH), and total hemolytic activity (CH50). Additionally, we quantified circulating immune complexes. We conducted general, stratified, correlation, and regression analyses.

**Results:**

Anti-Nucleocapsid sIgA was increased in post-COVID syndrome samples compared with COVID-recovered controls. Although CH50 levels were similar, we detected a reduced concentration of C3. The levels of C4 were decreased but not significantly. Interestingly, in recently reinfected fully vaccinated patients, serum anti-Nucleocapsid IgG showed a negative correlation with FH and CH50. No differences were found for the concentration of circulating immune complexes. Regression analysis indicated that C3 levels can discriminate patients from controls efficiently, and combining anti- Nucleocapsid sIgA and C3 levels yielded improved discriminatory power.

**Discussion:**

The elevated anti-Nucleocapsid sIgA levels found did not correlate with increased anti-Nucleocapsid IgG in serum, as expected from their different temporal dynamics. The reduced C3 levels may reflect ongoing complement activation and subsequent consumption, which might be potentiated by increments in serum of anti-Nucleocapsid antibodies produced after reinfections. In conclusion, our findings suggest that salivary anti-Nucleocapsid IgA and C3 consumption, which seems to be more subtle parameter than CH50, may serve as candidate biomarkers of post-COVID syndrome, requiring validation in independent cohorts. Furthermore, these results implicate the complement system as a key dysregulated component of the immune response contributing to the pathophysiology of post-COVID syndrome, thus potentially amenable to targeted therapies.

## Graphical Abstract

**Figure:**
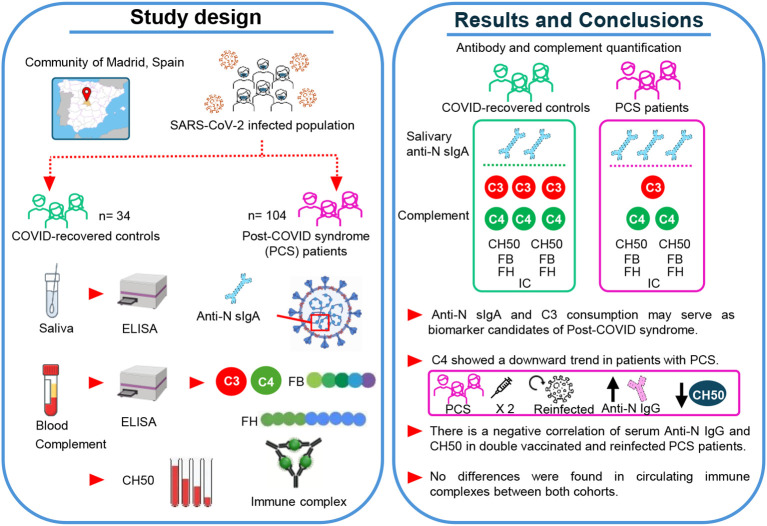
Study of salivary anti-Nucleocapsid secretory immunoglobulin A (sIgA), complement components (C3, C4), regulators (factors F and H), lytic activity (CH50) and circulating immune complex (IC) in COVID-recovered and post-COVID syndrome cohorts from Comunidad de Madrid (Spain). Image was produced using NIH Bioart illustrations.

## 1 Introduction

A substantial proportion of individuals recovering from acute COVID-19 subsequently experience persistent, often debilitating, post-acute sequelae collectively termed Long COVID, a designation originally introduced by patients. The World Health Organization (WHO) defines “post-COVID-19 condition” as the presence of symptoms that arise during or after a severe acute respiratory syndrome coronavirus 2 (SARS-CoV-) infection, persist for at least two months commonly becoming apparent around three months post-infection, and cannot be explained by an alternative diagnosis. More recently, the National Institute for Health and Care Excellence (NICE) updated this framework by distinguishing acute COVID-19 (up to 4 weeks), ongoing symptomatic COVID-19 (4–12 weeks), and post-COVID-19 syndrome (PCS), characterized by symptoms that persist for more than 12 weeks, and can last up to years (1). Recent analyses estimate that post-COVID-19 syndrome affected approximately 60–70 million people worldwide in 2021, with the overall population prevalence estimated to range from 2.9% to 10% across different countries (2, 3).

Although relevant progress has been made in the understanding of the physiopathology of the syndrome (4, 5), the dysregulation of the immune system is not yet completely understood (6). Within this framework, dysregulation of effector antibody responses and the complement system are emerging as a particularly compelling pathogenic mechanism contributing to the syndrome (7).

Immunoglobulin A (IgA) is the dominant antibody class in mucosal tissues and secretions, such as saliva, where it is referred to as secretory IgA (sIgA). sIgA plays an important role in pathogen neutralization while contributing to minimal inflammation (8). Notably, the detection of anti-Nucleocapsid (anti-N) sIgA has been used both to support the diagnosis of SARS-CoV-2 infection (9) and to assess immunoglobulin (Ig) responses following vaccination (10, 11). While some studies have established that anti-N IgG is elevated in patients with PCS (12, 13), considerably less is known about sIgA responses to the Nucleocapsid in this context.

The complement system, a cascade of plasma interacting proteins, is key in the lysis of pathogens and infected cells (14). It is activated through three major pathways. The classical pathway is initiated when C1 complex (each hexameric C1q plus two molecules of C1r and C1s serine proteases) binds to the crystallizable domain (Fc) of antigen-bound IgG or IgM, which causes a conformational change of the C1 complex and the activation of C1r and C1s tetramer. Then, C1r cleaves C1s, which in turn cleaves C4 and C2, leading to the formation of the C3 convertase (C4b2b) of the classical pathway, which process C3 into C3b (opsonin) and C3a (anaphylatoxin). The overall functional activity of the classical pathway can be assessed through the total hemolytic complement (CH50) assay, which measures the ability of a patient’s serum to lyse antibody-coated red blood cells.

The lectin pathway is activated mainly when pattern recognition molecules, such as mannose-binding lectin (MBL) or ficolins recognize specific carbohydrates on the surface of pathogens. It converges on the formation of the C3 convertase of the classical pathway. This pathway is primarily antibody independent.

On the other hand, the alternative pathway C3 convertase (C3bBb) is formed when spontaneously generated or surface-deposited C3b binds factor B (FB), a serine protease, which is then cleaved by another serine protease, factor D, releasing the small Ba fragment. The resulting C3bBb complex assembled on microbial surfaces efficiently cleaves additional C3, creating an amplification loop of complement activation. Properdin stabilizes the convertase and prolongs its half-life and functionality, while host regulators such as factor H (FH), factor I, MCP (membrane cofactor protein) and DAF (decaying accelerating factor) limit its formation on self-cells (14).

Specifically, FH binds C3b preventing the formation of the C3bBb convertase and acts as a cofactor for factor I, promoting proteolytic inactivation of C3b (iC3b), thereby protecting host cells from complement-mediated damage (14). FH is the most abundant regulator soluble in plasma, this together with the capacity of FH to function both in fluid phase and on specifically recognized host cellular surfaces, makes FH a key inhibitor of the alternative pathway and of the complement activation. It is therefore not surprising that many pathogens hijack FH to avoid complement action, including SARS-CoV-2 (15).

The activated complement cascade ultimately culminates in the formation of the membrane attack complex (MAC), which lyses target cells. In addition, the anaphylatoxins C3a and C5a recruit and activate immune cells, amplifying the immune response. C3b, and proteolyzed fragments iC3b or C3dg, deposited on the surfaces of pathogens, necrotic cells, or apoptotic cells can be recognized by complement receptors (such as CR1, CR2, CR3 and CR4) on phagocytic cells, which then mediate their phagocytosis (14).

Although the role of the complement during COVID-19 has been extensively studied (16–18), its contribution to PCS is not completely understood, while some studies reported increased levels of certain components such as C3 (19) in PCS patients, other reports did not find differences (20).

With respect to complement activation in post-COVID syndrome, one aspect that has received comparatively less attention is the potential pathogenic role of immune complex formation. These complexes may deposit in tissues, thereby promoting inflammation through activation of the complement cascade and recruitment and activation of immune cells (21).

We previously studied the antibody response of a PCS cohort in relation to a COVID-recovered cohort and found that PCS patients present a reduction in serum of total IgG, IgG1, IgG2 and IgG4 subclasses against the full-length Spike, while maintaining the IgG1 anti-RBD response. On the contrary, patients display a readily detectable IgG response to the Nucleocapsid, especially evident after a recent reinfection (13).

In this study we investigated salivary anti-Nucleocapsid secretory IgA (sIgA), a panel of selected complement factors (C3, C4, FB, FH), CH50 activity and circulating immune complexes. The primary aim was to compare salivary anti-N sIgA levels and concentrations of complement factors between individuals with post-COVID syndrome (PCS) and those who had recovered from COVID-19 without persistent symptoms. The secondary, exploratory aims were to assess the impact of vaccination and reinfection on these immunological parameters, and to examine correlations with previously measured immunoglobulins, including anti-N IgG, as well as with clinical symptoms. Finally, we performed regression analyses to explore the potential utility of salivary anti-N sIgA and C3 as candidate biomarkers for PCS.

## 2 Methods

### 2.1 Cohorts

**Figure 1:**
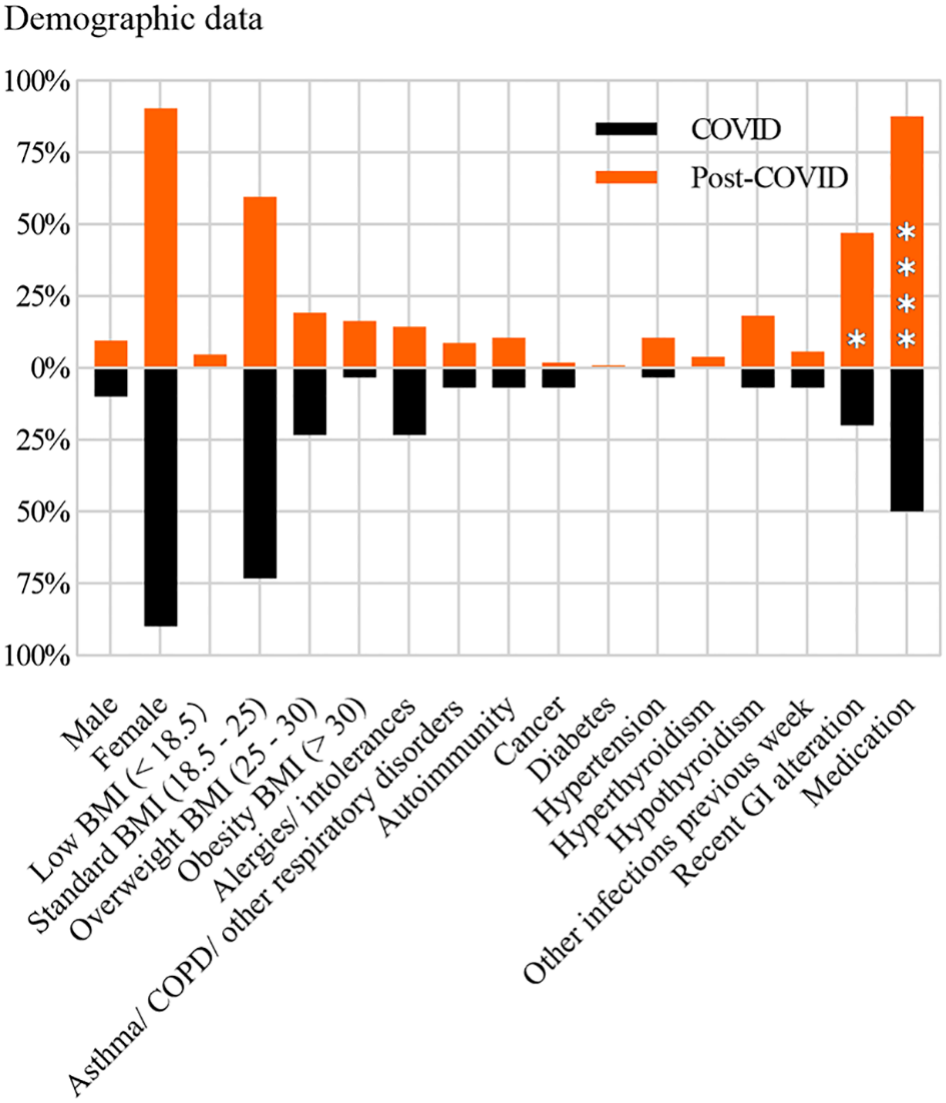
Demographic characteristics of the COVID-recovered and post-COVID syndrome cohorts. Sex distribution, body mass index (BMI), and comorbidities are shown. Differences in categorical variables between cohorts were assessed using the Chi-square or Fisher’s exact test, as appropriate. Asterisks indicate statistically significant differences between cohorts. **Figure 1** shows data adapted from **Supplementary Figure 2** published in the article Rossi et al., doi: https://doi.org/10.3389/fimmu.2025.1670324, under Creative Commons Attribution license (CC BY 4.0) (13).

Cohorts were previously described (13) and included 104 patients with “post-COVID” syndrome (PCS) and 34 samples from the COVID-recovered cohort, which comprised individuals who recovered from COVID-19 without any sequelae. Both cohorts were composed of 94- 95% woman of similar age: 51 with an interquartile range (IQR) of 44–59 for COVID and 48 with an IQR of 46–54 for PCS. Demographic characteristics are summarized in **Figure 1**. PCS and COVID recovered cohort do not show differences in gender, body mass index, and comorbidities such as allergies/intolerances, respiratory disorders, including asthma and chronic obstructive pulmonary disease (COPD). Other characteristics are described in (13), including information on date of infection, reinfections, vaccine types, number of vaccine doses and the interval between the last vaccination and sampling. To address differences in vaccine dose and reinfection status, we performed stratified statistical analyses (22), and fix the reinfection window to six-months.

### 2.2 Quantitation of IgA in saliva samples

A custom ELISA was performed to quantify secretory IgA in saliva against the SARS-CoV-2 Nucleocapsid (anti-N sIgA). Samples were analyzed in duplicate. Briefly, 96-well plates were coated overnight at 4°C with 0.2 μg of SARS-CoV-2 recombinant Nucleocapsid (Cat. CSB-EP3325GMY, Cusabio Technology LLC) dissolved in 100 μL per well of 0.05 M carbonate-bicarbonate buffer, pH 9.6 (Cat. C3041–50 CAP, Sigma-Aldrich), blocked, and then incubated with 1/10 diluted saliva samples alongside a commercial human serum IgA standard (Aeskulisa SARS-COV-2 NP IgA commercial kit, cat. 6121, Aesku diagnostics, Wendelsheim, Germany). The secondary antibody was a horseradish peroxidase (HRP)-conjugated goat anti-human IgA antibody (Cat. No. 34021, Fortis Life Sciences) used at 0.01 μg/mL. Five washes were performed between steps. Signals were developed using TMB substrate. The optical density (OD) at 450 nm with a reference wavelength of 620 nm was measured using an ELISA reader (SmartSpec 3000, Bio-Rad). The blank OD was subtracted from the sample readings, and the concentration was calculated using the equation of the standard curve fitted to a linear regression model.

### 2.3 Complement determinations

They were performed at the Complement Genetics and Molecular Analysis Laboratory at the CIB-CSIC center, directed by complement expert Santiago Rodríguez de Córdoba. Briefly, C3, C4, FH and FB were determined by sandwich ELISA, using antibodies generated in the laboratory. For C3, samples were measured in triplicate at two dilutions. Results are shown in mg/dL. Dynamic range: 1–100 ng/mL. Normal values in the laboratory: 70–150 mg/dL. For C4, samples were measured in duplicate at two dilutions. Dynamic range: 4–300 ng/mL. Normal values in the laboratory: 14–60 mg/dL. For FH: samples were measured in duplicate at two dilutions. Results are shown in μg/mL. Dynamic range: 2–140 ng/mL. Normal values in the laboratory: 90-285 μg/dL. For FB: samples were measured in duplicate at two dilutions. Results in μg/mL. Dynamic range: 15-990 μg/dL. Normal values in the facility: 75-280 μg/dL. CH50 hemolytic assay to determine total complement activity initiated by the classical pathway, was performed using rabbit IgG-coated erythrocytes sensitized with hemolysin. The assay was performed in triplicate using serum dilutions from 5-0.18% (dilutions 1/20 to 1/556). Results are expressed as the reciprocal serum dilution required to achieve 50% hemolysis (CH50), normalized to a control serum. Normal values in the facility range from 79 to 121%. The inter-assay variability was 6.83%, and the intra-assay variability was 6.76%.

Circulating immune complexes (IC) were quantified using a commercially available C1q immune complex binding ELISA kit (ref. DKO016, DiaMetra) according to the manufacturer’s instructions. Results were expressed as microgram equivalents per milliliter (μgEq/mL), where “Eq” denotes the amount of IC producing a signal equivalent to that generated by heat-aggregated human gamma globulin used as the assay calibrator, reflecting relative rather than absolute quantification.

### 2.4 Statistics

The strategy employed includes a general analysis followed by stratified analyses to account for the major factors conditioning the response, which are vaccine doses and reinfections (13, 22). Missing data were assumed to be missing at random, and no imputation or correction methods were applied, which represents a study limitation. We used GraphPad Prism software version 8.0.2. The normality of the distribution of variables was determined using the Shapiro–Wilk test. Continuous variables were compared using the Student’s t-test and Mann-Whitney U test, and analysis of variance (ANOVA) and the Kruskal-Wallis test for multiple comparisons when appropriate. Test used were two-sided and the significance level was set at α = 0.05. Pearsons’s coefficient (r_p_) or Spearman’s rank correlation coefficient (r_s_) were used to determine the correlation between variables as indicate on figures legends. P values were depicted as *P< 0.05; **P< 0.01; ***P< 0.001; and ****P< 0.0001.

Binary logistic regression and ROC curve analysis was performed using the GraphPad v8.0.2 and the R software (23). To ensure the robustness and predictive stability of the logistic regression model, influential observations were identified using Cook’s distance (Cook’s D). A predefined threshold of 4/n (where n is the sample size) was applied to detect outliers exerting disproportionate influence on parameters. Samples exceeding this threshold were excluded from the final ROC analysis.

## 3 Results

### 3.1 Analysis of anti-Nucleocapsid secretory IgA in saliva and complement factors in the serum from COVID and post-COVID syndrome individuals

**Figure 2:**
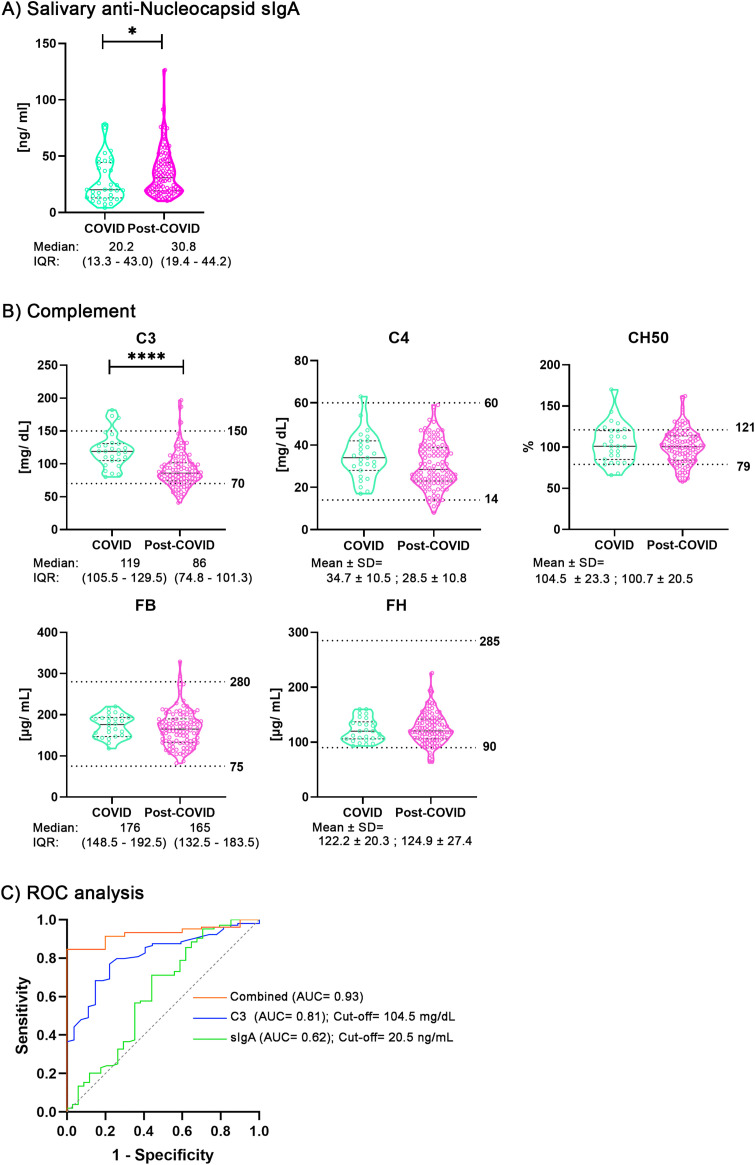
Comparison of salivary anti-nucleocapsid sIgA levels, serum complement levels, and hemolytic activity between COVID-recovered and post-COVID syndrome cohorts. **(A)** Comparison of the concentrations of salivary anti-N sIgA between COVID-recovered (n= 34) and post COVID syndrome (n= 104) samples. After assessing normality with the Shapiro-Wilk test, statistical significance was determined using the Mann–Whitney U test (*p < 0.05). **(B)** Serum concentrations of complement components (C3, C4, FB, and FH) and total hemolytic activity (CH50) were measured and compared between COVID-recovered (n= 27) and post-COVID (n= 104) cohorts. After assessing normality with the Shapiro-Wilk test, statistical significance was assessed using the Student’s t-test for C4, CH50 and FH, and the Mann–Whitney U test for C3 and FB, (****p < 0.0001). Data are presented in figures with the median and interquartile range (IQR). Complement normal values in the laboratory are indicated by dotted lines. **(C)** ROC analysis derived from binary logistic regression for the discrimination of post-COVID syndrome patients from COVID-recovered controls. Curves are shown in green for salivary anti-N sIgA, in blue for serum complement component C3, and in orange for the combined model (sIgA + C3). Calculated cut-offs are: sIgA 20.5 ng/mL (sensitivity 0.78, specificity 0.77); C3 104.5 mg/dL (sensitivity 0.56, specificity 0.71). AUC, area under the curve.

Demographic characteristics and comorbidities of the cohorts studied are shown in **Figure 1**. The cohorts showed statistically significant differences for recent gastrointestinal (GI) alteration (at the time of sampling) and medication use. Next, we determined the concentration of anti-N sIgA in saliva, and found a significant increase in PCS samples (**Figure 2A**). Since we had previously determined anti-N IgG in serum for the same cohort, we thought of analyzing a possible correlation between them, keeping in mind that in Spain vaccines are based on the Spike. As shown in **Supplementary Figure 1** we did not find any statistically significant correlation assessed by calculating Spearman’s correlation coefficient, which we interpreted to agree with earlier appearance of IgA and faster waning of this class immunoglobulin with respect to IgG (24).

Next, we analyzed the concentrations of complement factors C3, C4, FB and FH in serum and determined total hemolytic activity using the CH50 assay (**Figure 2B**). We found that C3 levels were statistically significant decreased in the post-COVID samples, indicating a possible consumption of this component. Likewise, the levels of C4 were lower in the PCS cohort than in the COVID-recovered cohort, but the difference did not reach a statistically significant difference. On the other hand, the levels of FB, FH and CH50 were similar between the two cohorts. Normal laboratory reference values are indicated by dotted lines in **Figure 2B**. **Supplementary Table 1** shows the number of samples falling outside these ranges, indicating that 18 (17.3%) PCS samples had values below the normal range for C3, whereas none of the COVID control samples did.

As previously mentioned, the cohorts studied exhibited statistically significant differences in the frequency of recent gastrointestinal alterations and medication use (**Figure 1** and (13)). Therefore, we assessed whether these variables could act as potential confounding factors for anti-N sIgA and C3. To this end, we included GI alteration and medication use in a multivariable regression model. The association between group and outcome remained essentially unchanged after adjustment, suggesting that these variables do not act as significant confounders for C3, but they seem to exert an effect on anti-N sIgA (**Supplementary Table 2**).

Interestingly, C3 consumption has been associated with specific PCS symptoms, such as fatigue (20). We therefore examined whether a similar association could be detected in the PCS cohort studied here, in comparison with the COVID-recovered cohort. We observed that patients experiencing fatigue exhibited greater C3 consumption in both cohorts. However, statistically significant differences were also observed between the fatigue and non-fatigue subsets (**Supplementary Figure 2**), these exploratory results suggest that while fatigue might be associated with C3 consumption to a certain degree, additional factors are likely involved. Furthermore, we investigated potential associations between C3 levels and the most frequently reported patient’s symptoms: headache, muscle pain, cough, anosmia, and join pain, and found no evidence supporting such associations (**Supplementary Table 3**).

Considering that the antibody response is altered in patients with post-COVID syndrome (13), together with reported antigen persistence (25), we hypothesized that circulating immune complexes may contribute to disease pathology. To address this, we quantified circulating immune complexes in our cohorts and found no differences (**Supplementary Figure 3**). Precisely, we detected only one positive individual in the COVID-recovered cohort and three in the PCS cohort, indicating that IC formation does not seem to be a general relevant pathogenic mechanism in PCS.

We next performed binary regression analysis to assess whether the increase sIgA and C3 consumption detected in PCS patients might be useful as possible biomarker candidates (**Figure 2C**). We found that AUC of sIgA shows low ability to discriminate PCS patients from controls (green, AUC = 0.62). The curve is closer to the diagonal, indicating limited predictive power. C3 shows good discriminatory ability (blue, AUC = 0.81). It is clearly above the diagonal line, indicating C3 alone is effective at distinguishing patients from controls. Combined model (orange, AUC = 0.93 (95%, CI: 0.88–0.98, P < 0.0001) shows excellent discriminatory performance. The curve is near the top-left corner, suggesting that combining sIgA and C3 improves prediction compared to either marker alone. Additionally, a cut-off for C3 was estimated at 104.5 mg/dL (at a sensitivity of 0.78 and a specificity of 0.77) (**Figure 2C**).

### 3.2 Effect of vaccination over complement levels and activity

**Figure 3:**
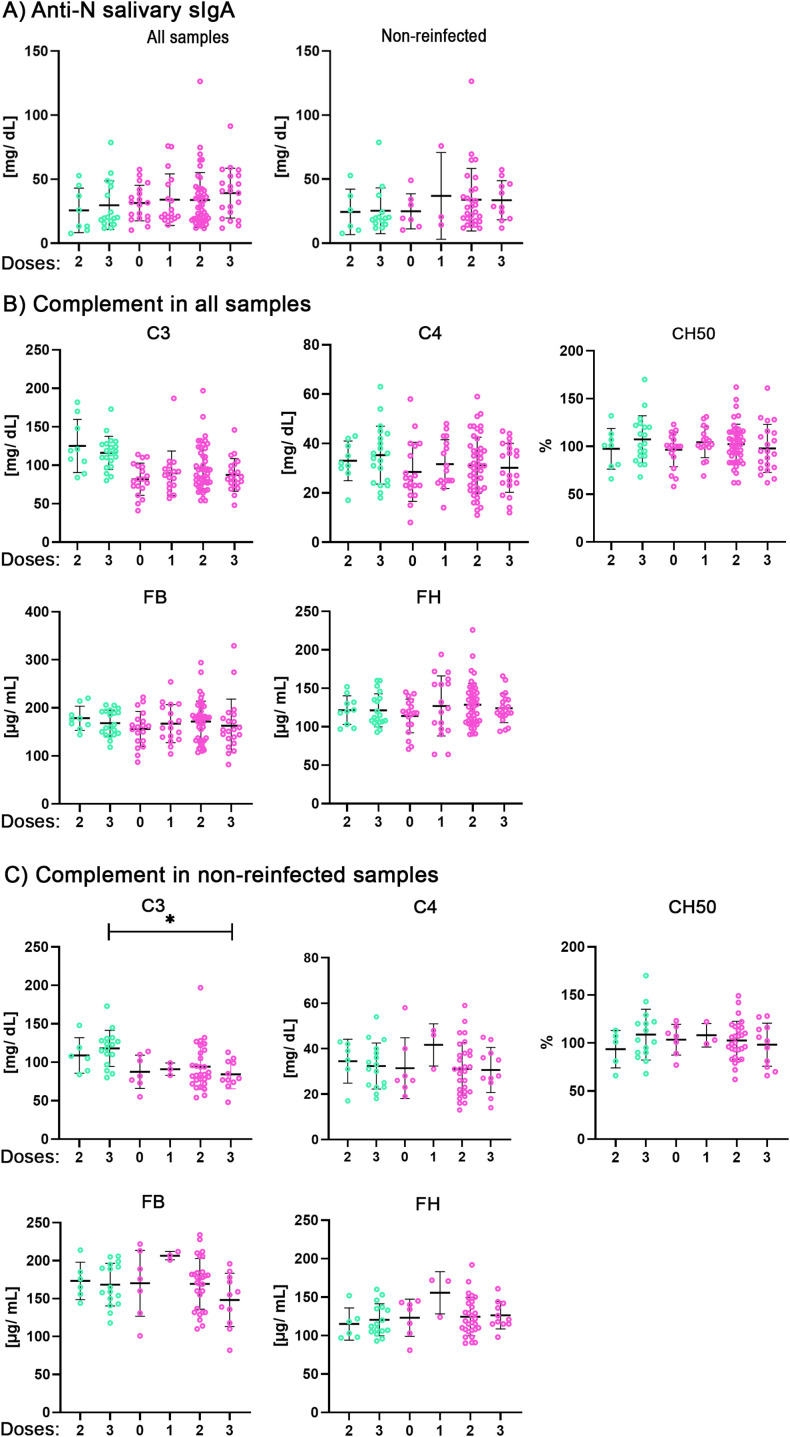
Comparison of salivary anti-nucleocapsid sIgA and serum complement levels in COVID-recovered and post COVID syndrome cohorts stratified by vaccine doses received and a recent reinfection. **(A)** Anti-Nucleocapsid sIgA in the vaccination subsets from the full cohort (left panel) and the non-reinfected subgroup (rightpanel). From left to right samples are: n: 8,17,19,17,47,21; non-reinfect n: 6,14,7,3,29,11. **(B, C)**. Serum concentrations of complement components **(B)** Full cohort. (C3, C4, FB, FH) n: 9,20,19,17,47,21 and total hemolytic activity (CH50) n: 8,19,19,17,47,21 **(C)** Non reinfected subgroup. (C3, C4, FB, FH) n: 6,16,7,3,29,11, CH50 n: 5,15,7,3,29,11, were measured in both cohorts, grouped by the number of vaccine doses received (0–3 doses). All groups were compared using Kruskal-Walli’s test followed by Dunn’s test. COVID-recovered samples are shown in green and post-COVID syndrome samples are shown in pink.

All vaccines used in Spain are based on the Spike. Vaccination influences the level of antibodies produced against the Spike, and these antibodies can activate the complement through the classical pathway (26). Therefore, we compared the levels of salivary anti-N sIgA (which might be indirectly influenced by vaccine doses) and the complement components, considering the vaccine doses administered to individuals in the two cohorts, including or not reinfections (**Figure 3**). We noticed that the level of C3 remained lower in post-COVID patients than in the COVID-recovered individuals, although statistical significance was only achieved when reinfections were not considered in individuals who received three vaccine doses.

Next, to visualize these findings, we generated paired plots of C3 and C4, as well as C4 and CH50 values, measured in the same individuals after two and three vaccine doses within each cohort (**Supplementary Figure 4**). Across cohorts, C3 and C4 levels exhibited a concordant pattern, with lower C3 concentrations generally corresponding to lower C4 concentrations, and vice versa. A similar relationship was observed between C4 and CH50, except for samples in the COVID-recovered group with 3 doses. Overall, the distributions and trends were comparable between the two cohorts.

### 3.3 Effect of infection over sIgA and complement levels and activity

**Figure 4:**
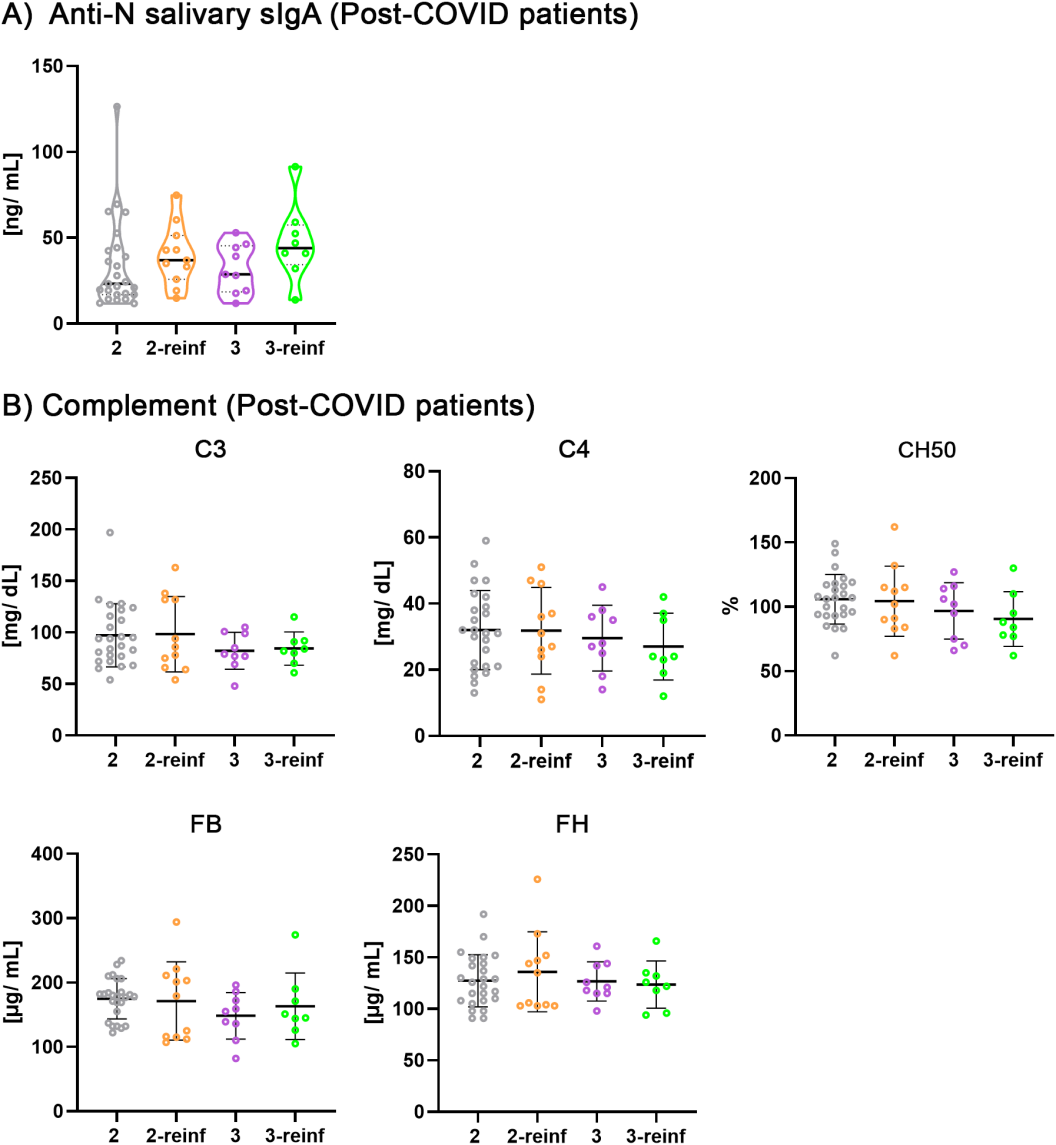
Comparison of salivary anti-nucleocapsid sIgA and serum complement levels in post COVID syndrome samples from patients infected before vaccination stratified by vaccination doses and a recent reinfection. **(A)** Salivary anti-N sIgA. **(B)** Serum concentrations of C3, C4, FB, FH complement components and total hemolytic activity (CH50) were measured. Samples used in the subsets, from left to right: n= 25, 11, 9, 8. All subsets were compared using Kruskal-Wallis test followed by Dunn’s test. Reinf, reinfection.

Next, we aimed to investigate the effect of a recent reinfection within the 6-months prior to sample collection; however, due to limitations in sample size, we analyzed only PCS samples from individuals who were infected prior to vaccination and subsequently received two or three vaccine doses (**Figure 4**), as previously done (13). Although no statistically significant differences were observed across the comparisons, a trend toward increased levels of salivary anti-N sIgA following reinfection can be perceived (**Figure 4A**). Any significant changes were detected for complement components and CH50.

### 3.4 Analysis of the correlation between the IgG anti-nucleocapsid and complement factors

Anti-Nucleocapsid antibodies can activate the classical complement pathway *in vitro* (27). Moreover, the nucleocapsid protein can reach adjacent non-infected cells and trigger complement-mediated lysis, thereby increasing nonspecific inflammation (27). In our cohort, PCS patients who were infected prior to vaccination and subsequently received two or three vaccine doses showed a marked increase in anti- N IgG levels following a recent reinfection (13). Based on these observations, we hypothesized that correlation analyses could provide insights into the mechanism explaining complement consumption; more specifically, whether increased anti-N IgG levels might be associated with a possible enhanced complement activation and consumption.

**Figure 5:**
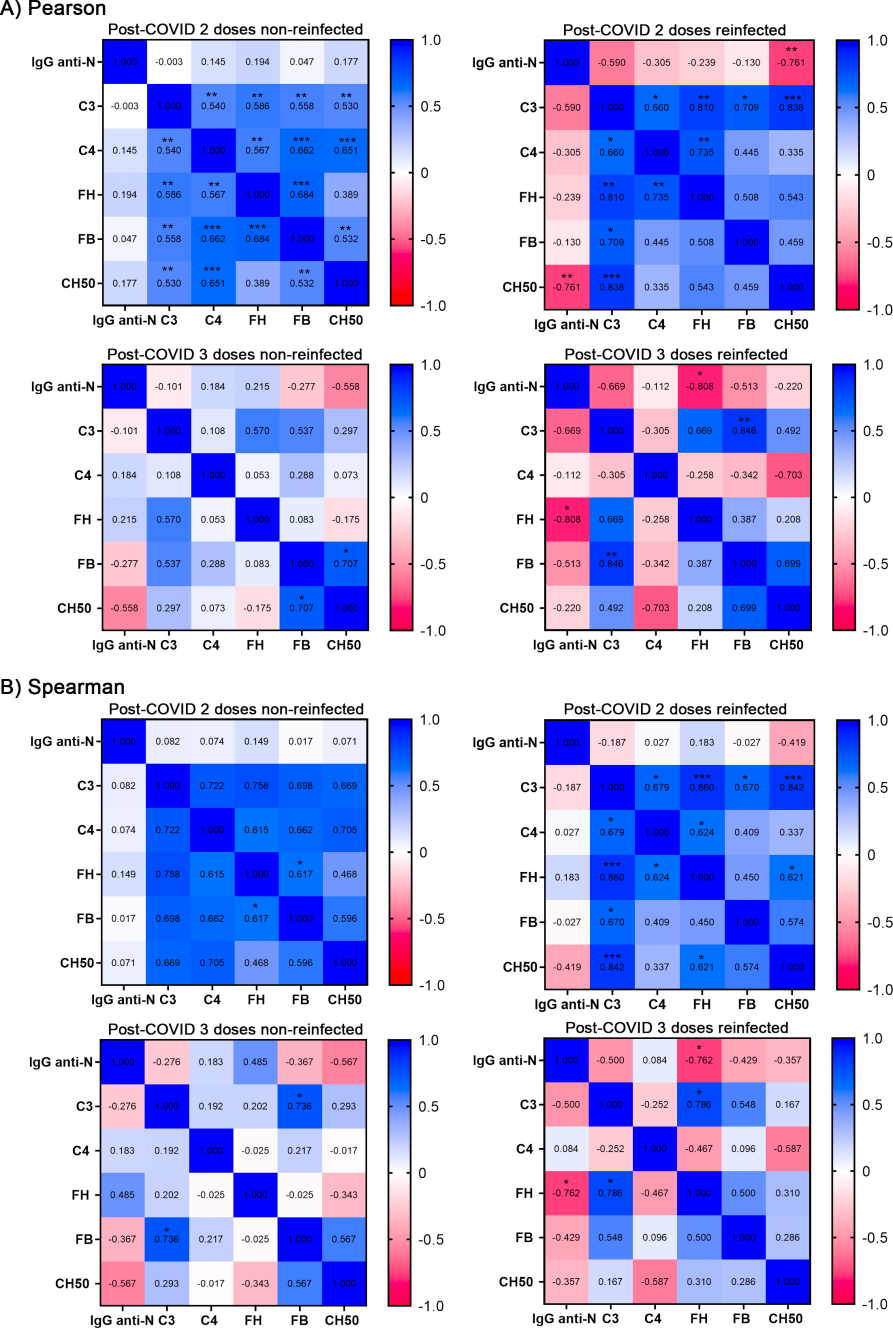
Correlation heatmaps of serum anti-nucleocapsid IgG and complement factors. Correlation analysis was performed to assess the relationships between serum anti-N IgG levels, complement factors (C3, C4, FH, FB), and CH50 activity. Heatmaps are shown for post-COVID syndrome patients with two or three vaccine doses, subdivided into non-reinfected and reinfected status groups (PCS-2 non-reinfected, n = 25; PCS-2 reinfected, n= 11; PCS-3 non-reinfected, n = 9; PCS-3 reinfected, n = 8). **(A)** Pearson’s correlation coefficients and **(B)** Spearman’s correlation coefficients are indicated in each cell and color-coded according to the scale bar. Statistical significance is shown with asterisks.

A general overview of the results (**Figure 5**) shows that various analyses in the reinfected subgroups changed towards a negative correlation (pink color), in agreement with a hypothetical increment in complement consumption after a recent reinfection. Specifically, we found a significant negative correlation between anti-N serum IgG and CH50 in patients with two doses and a recent reinfection (r*_p_*= - 0.761, p< 0.01), implicating that an increase in anti-N IgG value is associated with lower CH50 levels. However, we did not detect this association for anti-full-length Spike IgG, IgG1, IgG2 or IgG4, nor for anti-RBD IgG1, IgG2 and IgG4 (**Supplementary Figure 5**).

Interestingly, we detected a similar negative correlation with FH (r_p_= - 0.81, p< 0.01) in individuals with 3 vaccine doses, although the n for this analysis is not so large (n= 8). We interpreted that it could be due to the binding of FH to components of the virus such as the Nucleocapsid (15).

## 4 Discussion

Despite increasing clinical recognition, the immunological mechanisms driving post-COVID syndrome remain poorly characterized. We previously studied the antibody response of a cohort of PCS patients in comparison to a COVID-recovered cohort (13). We determined anti-N IgG in serum and focused on IgG subclasses (IgG1-IgG4), against the full-length Spike and the RBD, and performed stratified analysis considering vaccination doses and a recent reinfection (13). In the present study, using the same cohorts, although with some sample availability differences, we have determined salivary anti-N sIgA and relevant complement factors in the serum.

IgA is the predominant immunoglobulin at mucosal surfaces where it contributes to immune protection by neutralizing toxins, binding to pathogen surface components and blocking pathogen adherence, therefore at the end preventing epithelial invasion. Classical pathway activation by IgA via C1q binding is generally not efficient, as IgA lacks strong C1q interaction compared to IgG/IgM (28). Furthermore, IgA has been shown to modulate excessive immune responses during prolonged inflammation (29). These functions are especially critical in the context of respiratory viral infections (30), such as SARS-CoV-2 infection (31, 32).

SARS-CoV-2 has been shown to infect salivary glands and oral epithelial tissues, establishing a local environment capable of sustaining immune activation (33). However, the coronavirus can damage the mucosa and therefore alter the homeostasis of the immune response. As an example, the study of two post-COVID syndrome patients showed decreased production of the antimicrobial peptide Histatin-5, specifically produced by salivary glands, and sustained production of cytokines, among them interleukin (IL) IL17 (34). Interestingly, in severe COVID-19 patients, IL17 level in saliva can mirror its circulating level (35). Notably, IL17 promotes the recruitment and activation of neutrophils, which, as key effectors of innate immunity, might contribute to persistent inflammation through the release of proteolytic enzymes, pro-inflammatory mediators and extracellular traps; accordingly, a specific phenotypic neutrophil subpopulation has been described in hospitalized COVID-19 patients (36), and salivary proteomics analysis of convalescent patients showed increased neutrophil (myeloid) activation markers, such as myeloperoxidase (MPO) (37).

On the other hand, metabolomics studies of saliva from long COVID patients show that, at 45–60 days post-infection, there are changes in metabolite concentrations, such as lower abundance of creatine compared to healthy controls (38, 39). These findings suggest that the oral mucosa is altered and that its immune profile may partially reflect the systemic immune response (40). However, this relationship should be further investigated in depth in PCS patients.

Salivary secretory IgA (sIgA) directed against Spike have demonstrated greater sensitivity than sIgA against Nucleocapsid for detecting prior SARS-CoV-2 infection (41). However, because currently deployed vaccines in many countries, including Spain, are Spike-based, evaluating the mucosal immune response to the Nucleocapsid is appropriate to distinguish infection-induced from vaccine-induced immunity. Notably, in patients with post-COVID syndrome, emerging evidence reported by us and others suggests that the immune response to the Spike may be weaker than the response to the Nucleocapsid (13, 42, 43). Therefore, we analyzed salivary sIgA directed against the Nucleocapsid.

We observed significantly higher sIgA levels in the PCS cohort compared with the COVID-recovered cohort (**Figure 2A**), and found no correlation (**Supplementary Figure 1**) between salivary anti-N sIgA and previously determined serum anti-N IgG (13), which might be explained by the different temporal kinetics of mucosal and systemic responses after infection, as sIgA typically appears earlier than serum IgG (44). However, we evaluated recent GI alteration and medication use as possible confounders factors. The regression analysis shows that these variables seem to influence the outcome, although we cannot exclude that this happened because some samples could not be included in the analysis (unknown recent GI alteration and medication use, **Supplementary Table 2**).

Saliva samples obtained from COVID-19 patients, collected 20 to 90 days after initial clinical symptoms resolved (convalescent phase), showed that the concentration of anti-N IgA was higher in saliva than in plasma (37). These findings might indicate that salivary anti-N IgA is elevated from the acute phase to the post-COVID syndrome phase. In conclusion, our results raise the possibility that elevated salivary anti-N sIgA reflects persistent immune activation in patients with PCS, which might be partially attributable to Nucleocapsid antigenic persistence (25). Together, these findings support the hypothesis that PCS patients may display a dysregulated antibody-driven immune response, as previously suggested (45).

Complement dysregulation is considered a central contributing factor to hyperinflammation and thrombosis in COVID-19 and persistent immune activation in PCS (46, 47). Accumulating evidence indicates that all three complement pathways are hyperactivated in PCS (48). In our cohorts, we measured C3, C4, CH50, FB and FH (**Figure 2**). We found a statistically significant decrease in C3 in the PCS while C4 only showed a similar downward trend (**Figure 2**).

The observed decreased C3 levels suggests complement consumption and ongoing activation. This finding contrasts with a previous report of increased circulating C3 alongside T cell activation (19). This discrepancy may reflect differences in cohort characteristics, such as the timing of sampling, as that study included participants within three months post-infection (long COVID), whereas our cohort falls within post-COVID syndrome (PCS) phase.

Another exploratory study focusing on complement in PCS reported no significant differences of C3 levels (20), which may be attributable to its relatively small sample size (PCS n = 57) or other cohort characteristics, including gender distribution, as only 56% of PCS patients were women. Taken together, these findings support the notion of complement dysregulation in post-COVID syndrome, although the observed variability highlights the need for further investigation in future studies.

As mentioned, C3 can be consumed via the classical and lectin pathways, as well as through the alternative pathway and its amplification loop (14). To analyze whether C3 consumption, and the increased sIgA detected in PCS patients, might be useful as part of possible biomarker panel, we performed binary regression analysis (**Figure 2C**). ROC curves showed that C3 alone could discriminate PCS patients from controls (AUC=0.81). We determined a tentative C3 cut-off of 104.5 ng/mL, which requires validation in other cohorts. Although, salivary sIgA had limited predictive power (AUC=0.62), the combined model of sIgA and C3 exhibited excellent discrimination (AUC=0.93) and seems to benefit from a synergistic effect, suggesting that integrating mucosal and systemic immune markers enhances the ability to identify PCS patients. Therefore, we propose that C3 consumption may be more readily detectable than that of C4 and CH50. This suggests that C3 could contribute to the development of a potential biomarker within a clinical panel to aid patient stratification and follow-up. However, these findings require validation in further studies.

Clinically, PCS presents with heterogeneous but predominantly systemic symptoms, such as fatigue (49, 50). Previously, there were indications of a possible association between C3 levels and fatigue (20) which we studied in our cohorts but failed to detect (**Supplementary Figure 2**). We must keep in mind that fatigue is not a very specific symptom, which has also been associated with other aspects of PCS, e.g. Epstein-Barr Virus (EBV) reactivation (51). Additionally, the exploratory analysis of more frequent symptoms did not reveal any significant association with C3 levels (**Supplementary Table 3**).

Related to the C4 downward trend, C4 is associated with severity in the acute phase of COVID-19 (52). Morerover, a recent study reported that PCS patients present elevated levels of MASP-2/C1Inh (53), the covalently bound mannan-binding lectin-associated serine protease-2/C1inhibitor complex, which is normally elevated in plasma after infections, such as the one by SARS-CoV-2, and interestingly, it has been associated with COVID-19 severity (54). This complex is a subrogate marker of MASP-2 activation. MASP-2 is a serine protease that cleaves C4 into C4b and converts C4b-bound C2 and originates C4b2b, which is the classical/lectin pathway C3 convertase, that promotes C4 and C3 cleavage (55). These findings might help explain the C4 (and C3) decreased levels found in our PCS cohort.

Finally, no differences were detected for CH50, FB and FH in both cohorts (**Figure 2**). As stated, FH binds to C3b inhibiting complement activation. Although we did not observe statistically significant differences in FH levels between the COVID-recovered and PCS cohorts (**Figure 2**), recent molecular studies indicate that SARS-CoV-2 Nucleocapsid, but not the Spike, binds FH, among other complement regulatory proteins (15), perhaps contributing to complement imbalance.

Next, we analyzed the complement factors determined considering vaccine doses (**Figure 3**) and the trends obtained for the comparisons were like previous results (**Figure 2**). However, only when reinfections were not considered, did we observe a significant difference in C3 consumption (**Figure 3C**). We reasoned that the subset of non-reinfected individuals with three vaccine doses shows lower data dispersion that contribute to achieve statistical significance, confirming the results obtained in **Figure 2**. Additionally, the result may indicate a basal complement activation in PCS patients that is independent of a recent reinfection, which should be corroborated in other PCS cohorts. As noted in Methods, our cohorts were predominantly female (94 - 95%) of similar age (51 and 48 years old), which limited our ability to perform gender stratified analyses.

To visualize the relationship between C3 and C4 consumption, and how the latter influences the CH50 assay, we represented the values from the same individuals, subdividing them into two and three vaccine dose subsets (**Supplementary Figure 4**). We noticed that the general aspect was comparable between COVID and PCS subsets and that individuals with lower C3 presented also lower C4 (and *vice versa*), possibly indicating that C4 mediated complement activation (through classical or lectin mediated pathways) might contribute to C3 consumption, as previously interpreted (**Figure 2**). On the other hand, the consumption of C4 occurring through the classical/lectin pathways promotes terminal complex formation at the end increasing CH50 assay activity, which agrees with the result depiction (**Supplementary Figure 4**).

Getting infected by SARS-CoV-2 before vaccination is a risk factor for developing PCS (13, 56). Therefore, we analyzed salivary anti-N sIgA and complement factors in patients infected before vaccination in the PCS cohort, subdivided into two and three vaccine dose subsets (**Figure 4**). Unfortunately, due to the low number of individuals complying with this requirement, we could not include COVID-recovered samples in the analysis (study limitation). We found that while the complement analysis did not yield any statistically significant result, anti-N salivary sIgA was increased in the recently reinfected groups with two or three vaccine doses (**Figure 4A**), indicating that anti-N sIgA warrants further investigation as a marker for the detection of a recent reinfection in PCS patients.

Next, we analyzed the possible correlation between previously determined serum anti-N IgG (13) and the complement factors studied (**Figure 5**), in the same subsets as previously (**Figure 4**). Interestingly, we observed a negative correlation between serum anti-N IgG and CH50 (r*_p_*= - 0.761, P< 0.01), specifically in the two-dose vaccine group after recent reinfection, where the number of samples was higher than in three-dose reinfected group. Although not statistically significant, negative correlations were also found between serum anti-N IgG and C3 and C4. These results might indicate that PCS patients who respond to a new reinfection by increasing anti-N IgG (13) exhibit concomitant complement activation, reflected in presenting lower CH50 assay activity. However, these results should be interpreted with caution, as statistical significance was achieved only under a linear model (i.e., using Pearson’s correlation) and not with Spearman’s correlation analysis).

Remarkably, we did not detect this association for anti-full-length Spike IgG, IgG1, IgG2 or IgG4, nor for anti-RBD IgG1, IgG2 or IgG4 (**Supplementary Figure 5**). This is consistent with our previous observation that, with the exception of anti-RBD IgG4 (which is in general considered as an IgG not able to activate the complement), the levels of these immunoglobulins did not significantly increase following a recent reinfection in PCS patients who had received two or three vaccine doses within our PCS cohort (13). These findings are also in agreement with reports indicating that high titers of anti-Spike antibodies are required to detect complement activation *in vitro* (57).

In conclusion, we favor the hypothesis that antibodies against the Nucleocapsid may play a relevant role in potential chronic complement activation. Although the Nucleocapsid is primarily expressed intracellularly as part of the viral life cycle, it has also been detected on the surface of infected cells and, intriguingly, of uninfected cells (58). The Nucleocapsid binds electrostatically to heparan sulfate and heparin. Notably, heparan sulfate serves as a binding site for Factor H (FH), which protects host cells from complement activation (14) and also functions as a cellular attachment factor for SARS-CoV-2 (59). We speculate that increased deposition of Nucleocapsid on the cell surface could displace FH, thereby facilitating activation of the alternative pathway. Concurrently, high levels of surface-associated Nucleocapsid may promote the binding of anti-N IgG antibodies, further enhancing complement activation by the classical pathway.

The hypothesis of ongoing activation of the classical complement pathway through recognition of antigens expressed on the cell surface (e.g. Nucleocapsid), bound by their specific antibodies (anti-N IgG), is further supported by the absence of detectable circulating immune complexes in PCS samples (**Supplementary Figure 3**).

Additionally, the Nucleocapsid, but not the Spike, binds to various complement regulatory proteins (CRPs), such as C1-INH, C4 binding protein and FH (15), although we did not detect any change in the latter. Nucleocapsid bound to these CRPs may promote their degradation by fixing anti-N antibodies which would activate the complement. In this context, increased levels of anti-N IgG serum antibodies may critically influence complement system regulation, as we preliminary observed in the correlation analyses (**Figure 5**).

To end, the reduced circulating C3 levels found in PCS patients support the hypothesis of persistent complement activation beyond the acute phase of SARS-CoV-2 infection. Ongoing activation of the complement cascade may lead to sustained cleavage and consumption of C3, generating bioactive fragments such as C3a, and downstream effectors including C5a, and the membrane attack complex (C5b-9) (48). These mediators are known to promote endothelial activation, leukocyte recruitment, platelet aggregation, and tissue factor expression, thereby fostering a pro-inflammatory and pro-thrombotic microenvironment in patients with PCS (42, 48).

Salivary IgA primarily reflects mucosal immunity in the upper airway, whereas complement components such as C3 are key mediators of systemic innate immunity in blood and endothelia. To date, mucosal markers such as salivary anti-Nucleocapsid IgA remain underexplored in post-COVID syndrome. Therefore, our study indicates that investigating both mucosal and systemic immune parameters may represent a valuable approach.

In conclusion, despite the limitation that our cohorts are predominantly composed of woman and certain constraints related to the size of the COVID-recovered control cohort, our study shows that post-COVID syndrome patients present increased levels of salivary anti-Nucleocapsid sIgA and a clear decrease in complement C3 level. We favor the interpretation that both findings most likely do not reflect a direct mechanistic link. On the other hand, our results do indicate a potential association between serum anti-Nucleocapsid IgG and CH50, which warrants further investigation. While this finding should be corroborated in further studies, binary logistic regression analysis indicates that anti-N sIgA and C3 could potentially serve as synergistic candidate biomarkers of immune system dysregulation in post-COVID syndrome.

## Data Availability

The raw data supporting the conclusions of this article will be made available by the authors, without undue reservation.
