# Supplementary material for: Increased anti-nucleocapsid secretory IgA and consumption of complement component 3 in post-COVID syndrome patients
Source: Front Immunol. 2026 May 14;17:1822171. doi: 10.3389/fimmu.2026.1822171 (PMC13216748; doi:10.3389/fimmu.2026.1822171)
Supplement: Supplementary file 1 [file DataSheet1.pdf]

Supplementary Material

1 Supplementary Figures

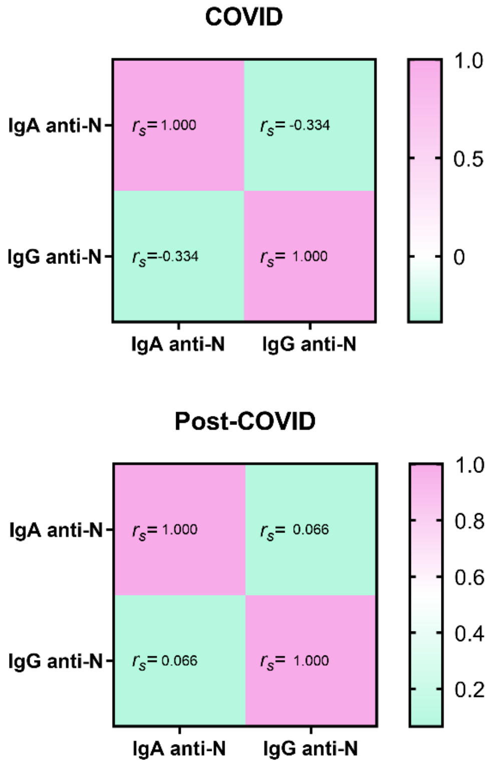

Supplementary Figure 1. Correlation of anti-Nucleocapsid secretory IgA and serum anti-Nucleocapsid IgG in COVID-recovered and post-COVID syndrome cohorts. Spearman correlation matrices comparing the levels of salivary anti-N sIgA with previously measured serum anti-N IgG within COVID (n= 25) and post COVID cohorts (n= 104).

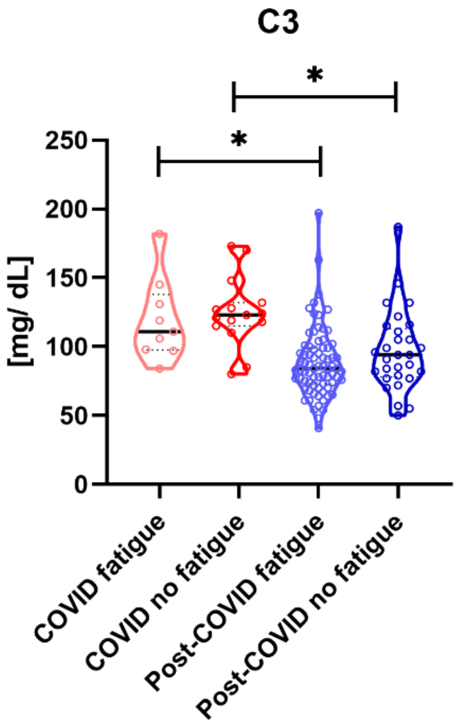

Supplementary Figure 2. Serum complement C3 levels stratified by the presence or absence of fatigue in COVID-recovered and post-COVID syndrome cohorts. Serum C3 concentrations were measured in participants and divided according to the presence or absence of fatigue. Data are presented with the median and interquartile range. Statistical analyses were conducted using Kruskal-Wallis test followed by Dunn´s multiple comparisons test. Sample sizes from left to right are: n= 9, 15, 75, and 29.

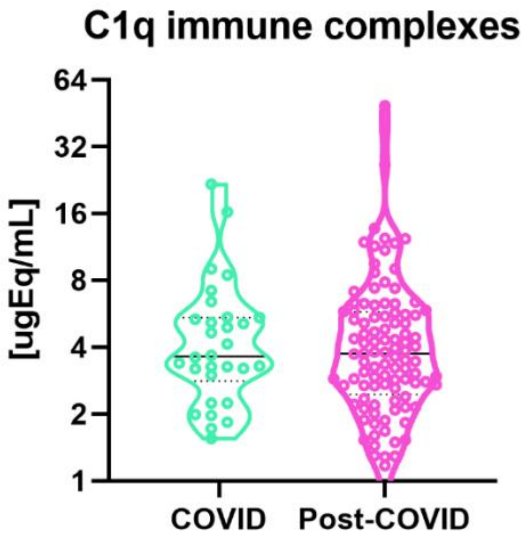

Supplementary Figure 3. Serum levels of C1q immune complexes in COVID-recovered and post-COVID syndrome cohorts. Serum concentrations of C1q immune complexes were measured and compared between COVID-recovered (n= 30) and post-COVID syndrome (n= 104) cohorts. Statistical significance was assessed using the Student´s t-test.

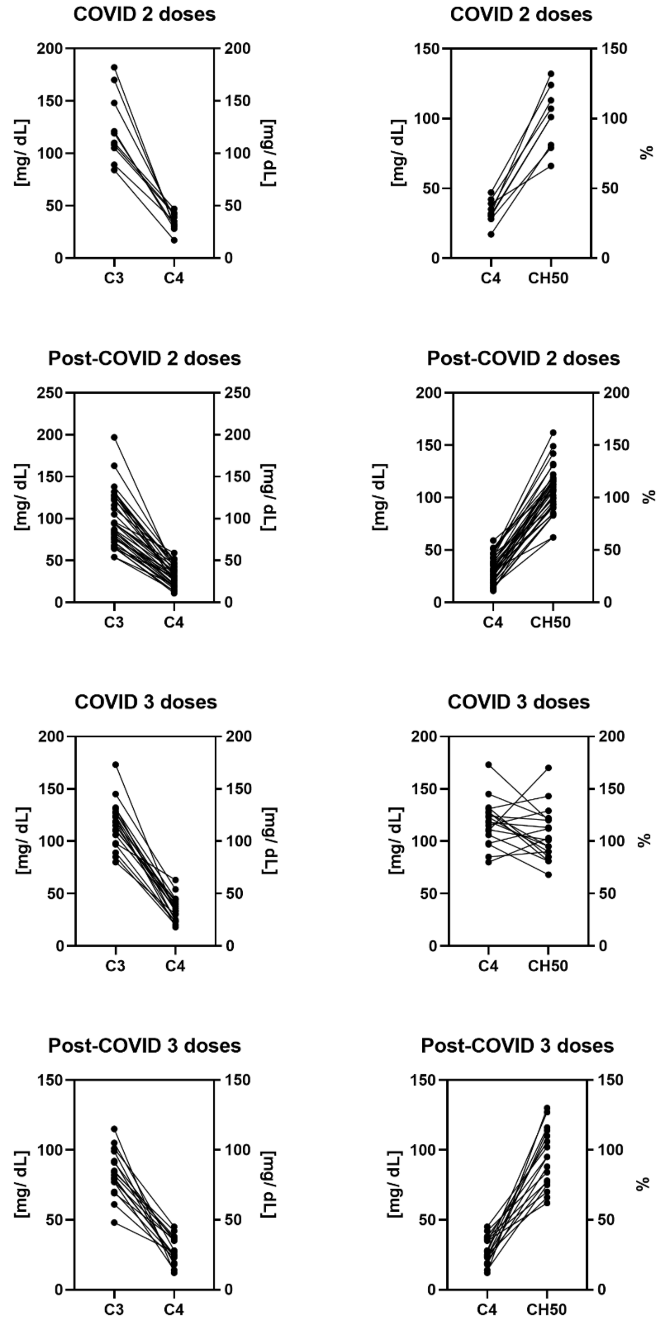

Supplementary Figure 4. Paired representations of C3 and C4 complement components, and C4 and hemolytic activity (CH50) within COVID-recovered and post COVID cohorts stratified by two and three vaccination doses. Sample sizes are: for C3–C4 analysis, COVID 2 doses (n= 10), COVID 3 doses (n= 19), PCS 2 doses (n= 36), and PCS 3 doses (n= 17); for C4–CH50 analysis COVID 2 doses (n= 9), COVID 3 doses (n= 18), PCS 2 doses (n= 36), and PCS 3 doses (n= 17).

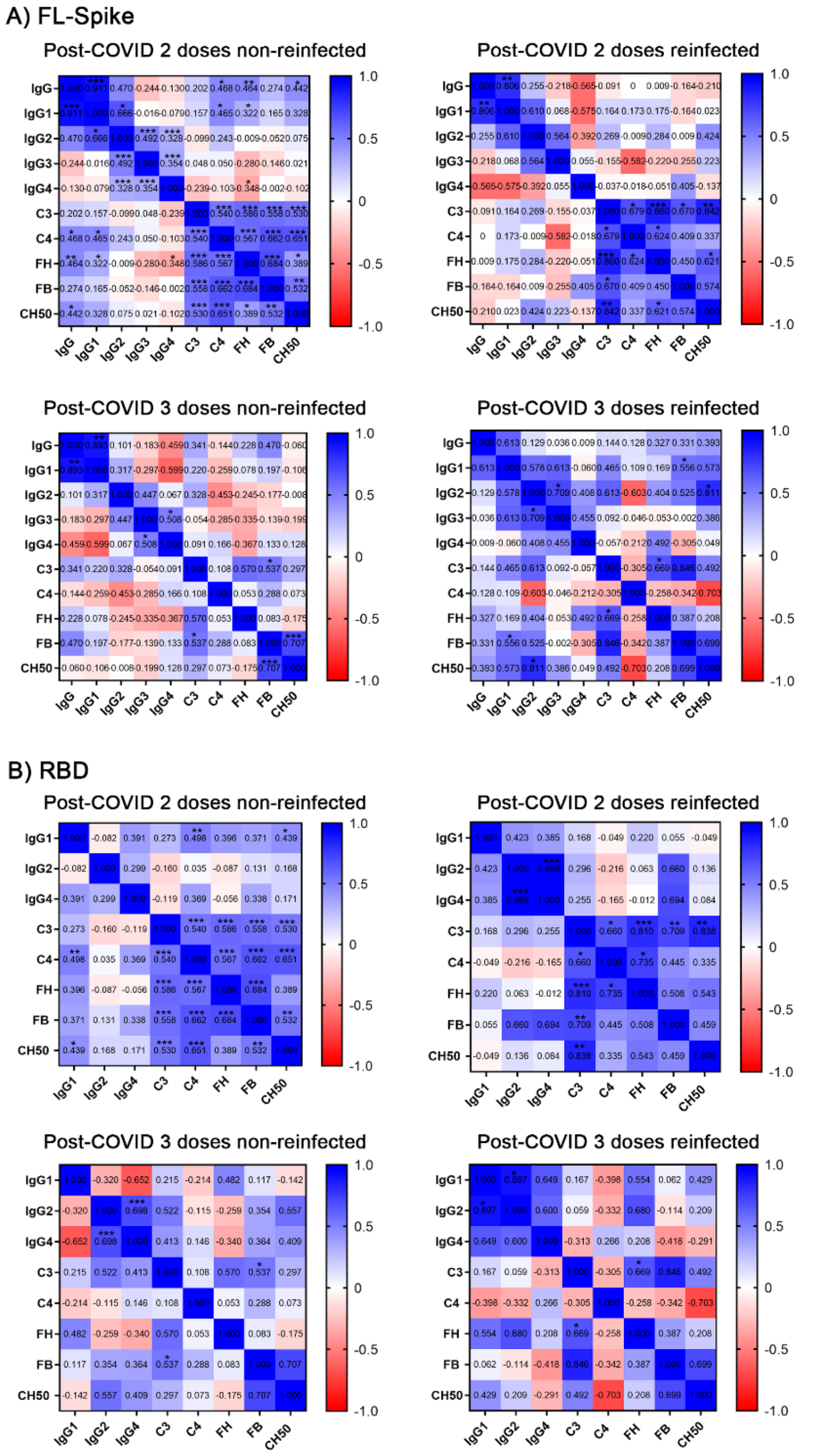

Supplementary Figure 5. Correlation heatmaps of immunoglobulins and complement factors. Correlation analysis was performed to evaluate the relationships between immunoglobulins and complement factors (C3, C4, FH, FB) as well as CH50 activity for post-COVID syndrome (PCS) patients who received two or three vaccine doses and were further stratified according to their reinfection status (PCS-2 non-reinfected, n= 25; PCS-2 reinfected, n= 11; PCS-3 non-reinfected, n= 9;

PCS-3 reinfected, n= 8). **A)** Anti-full-length (FL)-Spike IgG correlations **B)** Anti-RBD correlations. Spearman's correlation coefficients are indicated in each cell and color-coded according to the scale bar. Statistical significance is shown with asterisks.

2 Supplementary Tables

Supplementary Table 1. Number of samples with complement component levels outside the laboratory reference range in the COVID-recovered (COVID) and post-COVID syndrome (PCS).

		**C3** (70 - 150 mg / dL)	**C4** (14 - 60 mg / dL)	**FH** (90 - 285 µg / dL)	**FB** (75 - 280 µg / dL)	**CH50** % (79 - 121 %)
**COVID** (n = 27)	Total Out	3 (11.1%)	1 (3.7%)	0 (0%)	0 (0%)	8 (29.6%)
	Higher	3 (11.1%)	1 (3.7%)	0 (0%)	0 (0%)	6 (22.2%)
	Lower	0 (0%)	0 (0%)	0 (0%)	0 (0%)	2 (7.4%)
**PCS** (n = 104)	Total Out	21 (20.2%)	4 (3.8%)	5 (4.8%)	2 (1.2%)	29 (27.9%)
	Higher	3 (2.9%)	0 (0%)	0 (0%)	2 (1.9%)	14 (13.5%)
	Lower	18 (17.3%)	4 (3.8%)	5 (4.8%)	0 (0%)	15 (14.4%)

Supplementary Table 2. Multivariable linear regression analysis of possible confounding factors for sIgA and C3 levels.

**Variable**	**β (95% CI)**	**P value**
**sIgA**		
Group (PCS n = 104 vs. COVID n = 25)	4.20 (-5.12 to 13.52)	0.37
GI alteration (Yes n = 52 vs. No n = 77)	2.93 (-4.10 to 9.96)	0.41
Medication use (Yes n = 104 vs. No n = 25)	3.14 (-6.00 to 12.27)	0.50
**C3**		
Group (PCS n = 104 vs. COVID n = 27)	-28.80 (-41.23 to -16.37)	**< 0.0001**
GI alteration (Yes n = 52 vs. No n = 79)	-0.70 (-10.36 to 8.96)	0.89
Medication use (Yes n = 104 vs. No n = 27)	-2.00 (-14.15 to 10.15)	0.75

GI: gastrointestinal

Supplementary Table 3. Association of post-COVID syndrome symptoms with C3 levels.

C3	COVID	Post-COVID	P	P*c*
**Headache**	115 (104,129.25)	83 (76,101)	0.0199	0.1194
	4（18.1%）	57（54.8%)		
**Muscle pain**	144 (125,163)	87.5 (78,103.5)	0.061	0.366
	2 (9%)	54 (51.9%)		
**Cough**	102 (84.5,163)	88 (74,105)	0.233	1
	4（16.7%）	47 (45.2%)		
**Anosmia**	146.5 (111,182)	84 (77.5,99.5)	0.041	0.246
	2 (8.3%)	37 (35.6%)		
**Joint pain**	144 (106,182)	95 (78,111)	0.131	0.786
	2 (8.3%)	31 (29.8%)		

The first rows show median and range values. The second rows show number and percentages (%). Statistical analysis was performed using the Chi-square test or Fisher exact test. Corrected p values (Pc) were obtained using Bonferroni's correction.
